# What determines host specificity in hyperspecialized plant parasitic nematodes?

**DOI:** 10.1186/s12864-019-5853-4

**Published:** 2019-06-06

**Authors:** Michael Sabeh, Etienne Lord, Éric Grenier, Marc St-Arnaud, Benjamin Mimee

**Affiliations:** 1 St-Jean-sur-Richelieu Research and Development Center, Agriculture and Agri-Food Canada, St-Jean-sur-Richelieu, Quebec Canada; 2 Biodiversity Center, Institut de recherche en biologie végétale, Université de Montréal and Jardin botanique de Montréal, Montreal, Quebec Canada; 3 INRA, UMR1349 IGEPP (Institute of Genetics, Environment and Plant Protection), F-35653 Le Rheu, France

**Keywords:** Plant-parasitic nematode, Globodera, Transcriptomic study, RNA-seq, Pathogenicity, Effectors, Host range

## Abstract

**Background:**

In hyperspecialized parasites, the ability to grow on a particular host relies on specific virulence factors called effectors. These excreted proteins are involved in the molecular mechanisms of parasitism and distinguish virulent pathogens from non-virulent related species. The potato cyst nematodes (PCN) *Globodera rostochiensis* and *G. pallida* are major plant-parasitic nematodes developing on numerous solanaceous species including potato. Their close relatives, *G. tabacum* and *G. mexicana* are stimulated by potato root diffusate but unable to establish a feeding site on this plant host.

**Results:**

RNA sequencing was used to characterize transcriptomic differences among these four *Globodera* species and to identify genes associated with host specificity. We identified seven transcripts that were unique to PCN species, including a protein involved in ubiquitination. We also found 545 genes that were differentially expressed between PCN and non-PCN species, including 78 genes coding for effector proteins, which represent more than a 6-fold enrichment compared to the whole transcriptome. Gene polymorphism analysis identified 359 homozygous non-synonymous variants showing a strong evidence for selection in PCN species.

**Conclusions:**

Overall, we demonstrated that the determinant of host specificity resides in the regulation of essential effector gene expression that could be under the control of a single or of very few regulatory genes. Such genes are therefore promising targets for the development of novel and more sustainable resistances against potato cyst nematodes.

**Electronic supplementary material:**

The online version of this article (10.1186/s12864-019-5853-4) contains supplementary material, which is available to authorized users.

## Background

Nematodes are a very diverse phylum of animals living in a wide range of environments. Most of them feed on microorganisms or organic matter detritus in a free-living mode of existence. However, some species have diverged towards a parasitic lifestyle on higher organisms such as plants and animals, often in complex obligate associations. This transition to parasitism has followed morphological adaptation, but also the acquisition of genes coding for excreted proteins giving them the ability to feed and survive on their host. Different human- and animal-parasitic nematodes have been studied extensively but despite the importance of plant-parasitic nematodes (PPN), many aspects of the infection process are still largely unknown [1]. PPNs are plant pathogens of great importance and represent a significant constraint on global food production causing yield losses estimated at $157 billion every year [2]. Over 4100 species of PPNs have been described to date growing on all major cultivated crops in the world, the most damaging families being the *Meloidogynidae* and *Heteroderidae* including *Meloidogyne* spp., *Globodera* spp., and *Heterodera* spp. [1, 3]. It is now generally accepted that after the development of an anatomical structure used for plant cell wall puncturing and nutrient uptake called the stylet, the ability of nematodes to parasitize plants was facilitated by the acquisition of bacterial genes through horizontal gene transfer (e.g. cellulases, pectate lyases, xylanases, galactosidases, and expansin-like proteins) [4–9]. Host penetration, the establishment of a feeding site and suppression of host defenses are key stages of the infection process and are highly dependent on their set of specific secreted proteins called effectors, used by PPNs to manipulate the host to their benefit [10]. These effectors are responsible for most of the interactions with their host and given their importance in the infection mechanisms, substantial research efforts have been put on these molecules in recent years [11]. These effectors are also typically involved in evolutionary arm races between plants and parasites [12]. Although different plant-parasitic nematodes within a group (e.g. root-knot nematodes, cyst nematodes) have a common arsenal of effectors, it is not yet known exactly how this arsenal differs among species and especially among closely related species showing different host ranges. It was previously showed that the sequence polymorphism of the pectate lyase 2 effector among two potato cyst nematode species and one tobacco cyst nematode species can be associated to some extent to their host range variation [13]. Also at an intraspecific level, the ability to grow on potato cultivars harboring the H1 resistance gene was concordant with polymorphism in two effector genes (putative cellulose binding protein and 3H07 ubiquitin extension) found in virulent *Globodera rostochiensis* populations [14]. Studies conducted on other plant parasites like fungi have also associated variants in effector genes with their host range variation, as shown by the specific non-synonymous variants in two genes that appeared to be crucial for *Zymoseptoria tritici* virulence on wheat [15]. It has also been shown that effector genes contain a greater proportion of non-synonymous mutations compared to other genes [14]. To better understand this relationship, a formal comparison of genetic variation between closely related species having different host ranges would help to identify elements that are associated with host specificity.

The genus *Globodera* includes more than a dozen species parasitic to either Solanaceae or Compositae plants, which can be differentiated through their host range. All *Globodera* of *Solanaceae* species are parasitic on tomato and some *solanaceaeous* weeds but only *G. rostochiensis* (Wollenweber), *G. pallida* (Stone)*,* and *G. ellingtonae* (Handoo, Carter, Skantar & Chitwood) are parasitic on potato. *G. tabacum* (Lownsbery & Lownsbery) is found in a dozen tobacco-producing countries [16] and is parasitic on tobacco but not on potato, while *G. mexicana* (Campos-Vela) is mainly found in Mexico [17] and is parasitic on *Solanum nigrum* but not parasitic on either tobacco or potato.

Potato cyst nematodes (PCN), *G. rostochiensis* and *G. pallida*, are hyperspecialized plant-parasites considered as major agricultural threats as they are responsible for the loss of 9% of the world’s potato production each year [16, 17]. Originated in South America, PCN are now present in over 75 countries, where they are often considered as regulated quarantine organisms [18, 19]. The PCN infection process starts when the dormant eggs receive an appropriate chemical signal from the root diffusate of a potential host. The nematodes will then hatch and migrate toward the roots. Using different secreted enzymes, they will enter the roots and transform a plant cell in the root inner cortex layers to establish a complex feeding site, named syncytium, a highly metabolically active structure with enriched cytoplasm [18, 19]. The PCN induce a cascade of changes in host-gene expression, and cell fusion to form a syncytium. How the effectors cause the hypertrophy and endopolyploidization of feeding cells and their interplay with plant hormones is not yet fully understood [8, 20]. The important diversion of plant nutriments towards the nematodes will limit plant growth and eventually cause heavy yield loss. After maturation and fecundation, PCN females will dry to form a cyst, a protective shell, containing up to 300 eggs able to survive more than 20 years in soil [21, 22].

As *G. tabacum* shares a high level of genetic similarity with *G. rostochiensis* [13, 23], and *G. mexicana* with *G. pallida* [24, 25], these species are therefore perfect candidates for a transcriptomic comparison analysis according to their pathogenic status on potato plants. Transcriptomic study allows reduction of genomic complexity by sequencing only coding regions, in which a large proportion of significantly functional variants are expected, also allowing identification of genes whose expression or allelic frequency can be correlated to a specific characteristic [26]. Other studies have focused on the discovery of effectors in plant-parasitic nematodes, including PCNs [27–31], as well as on the transcriptomic study of different pathotypes [14] or life stages [32, 33] of *G. rostochiensis* and *G. pallida*, but to our knowledge, a direct transcriptomic comparison of pathogenic and non-pathogenic *Globodera* species on potato has never been done. The aims of this study were to characterize the transcriptomic differences between four *Globodera* species exposed to potato root diffusate and to identify genes putatively involved in host specificity, using RNA sequencing to look at changes in gene expression and genetic variation between populations.

## Results

### Sequencing and mapping

Exposure to potato root diffusate successfully induced hatching of a similar proportion of J2 larvae for all samples (data not shown). RNA sequencing of eight *Globodera* populations yielded a total of 233 M paired-end reads (2 × 125 bp). A mean of 29 M reads per sample, spanning from 24.1 M to 36.2 M reads was obtained (Table 1). The percentage of reads that successfully mapped to the *G. rostochiensis* reference transcriptome was on average 79.4% for *G. rostochiensis*, 74.2% for *G. pallida*, 69.3% for *G. tabacum* and 56.1% for *G. mexicana*. Horizontal coverage (breadth of coverage) was similar for all populations with reads mapping to 95.9 to 98.9% of the reference transcripts (Table 1). The phylogenetic analysis, performed using the small subunit ribosomal RNA gene, resulted in a greater genetic similarity between *G. rostochiensis* and *G. tabacum*, and between *G. pallida* and *G. mexicana* (Additional file 1: Figure S1).

**Table 1:** Sequencing yield and mapping statistics

Sample ID	Sequenced Reads (M)	Mapped (%) ^a^	Reference coverage (%) ^a^	Total variants
GrQC	36.2	81.74	98.90	67,996
GrU1	28.1	77.02	98.42	68,778
GpA5	30.8	71.65	97.78	639,271
GpB1	24.1	76.73	97.26	608,293
GtA1	29.1	81.54	96.42	383,996
GtA2	27.5	57.01	95.92	365,750
GmA1	28.5	71.90	97.39	537,306
GmA2	28.2	40.25	96.75	500,035

^a^Reads were mapped on *Globodera rostochiensis* reference transcriptome (nGr.v1.1)

### Quantitative analysis and identification of differentially expressed genes

Seven transcripts were unique to *G. rostochiensis* and *G. pallida* and presumed missing from *G. mexicana* and *G. tabacum* transcriptomes (Table 2). Among these, six were coding for unknown proteins, and the remaining one (GROS_g11284.t1) was annotated as Polyubiquitin-B protein. To further investigate their functions, transcripts of unknown proteins were realigned to *G. rostochiensis* transcriptome to find similar sequences. Transcript GROS_g12023.t1, was found to be similar to GROS_13581.t1, which have a CHROMO domain (PFAM 00385), suggesting those genes could be implicated in the modification of the chromatin organization. In order to investigate if these transcripts correspond to missing genes in the *G. tabacum* and *G. mexicana* species, qPCR validation were performed on genomic DNA of the four *Globodera* species. Amplification products were obtained in all cases, except that amplification product corresponding to GROS_g11284.t1 and GROS_g09749.t1 were not detected for *G. mexicana* and amplification product corresponding to GROS_g12023.t1 was not detected for either *G. tabacum* and *G. mexicana*, suggesting in this last case a complete absence of the corresponding gene in the *G. mexicana* and *G. tabacum* genomes.

**Table 2:** Sequences only expressed in PCN species (*Globodera rostochiensis* and *G. pallida*)

SeqID	Gene Description	Entry number (Organism) ^a^
GROS_g09749.t1	Unknown	A0A183CCS7 (*Globodera pallida*)
GROS_g10809.t1	Unknown	A0A1I7SMH2 (*Bursaphelenchus xylophilus*)
GROS_g11284.t1	Polyubiquitin-B-like	A0A183CCZ8 (*Globodera pallida*)
GROS_g12023.t1	Unknown	A0A183C0B5 (*Globodera pallida*)
GROS_g13375.t1	Unknown	A0A183C870 (*Globodera pallida*)
GROS_g13474.t1	Unknown	A0A183BU61 (*Globodera pallida*)
GROS_g13669.t1	Unknown	A0A0K6FY64 (*Rhizoctonia solani*)

^a^Relates to UniProt database

**Fig. 1:**
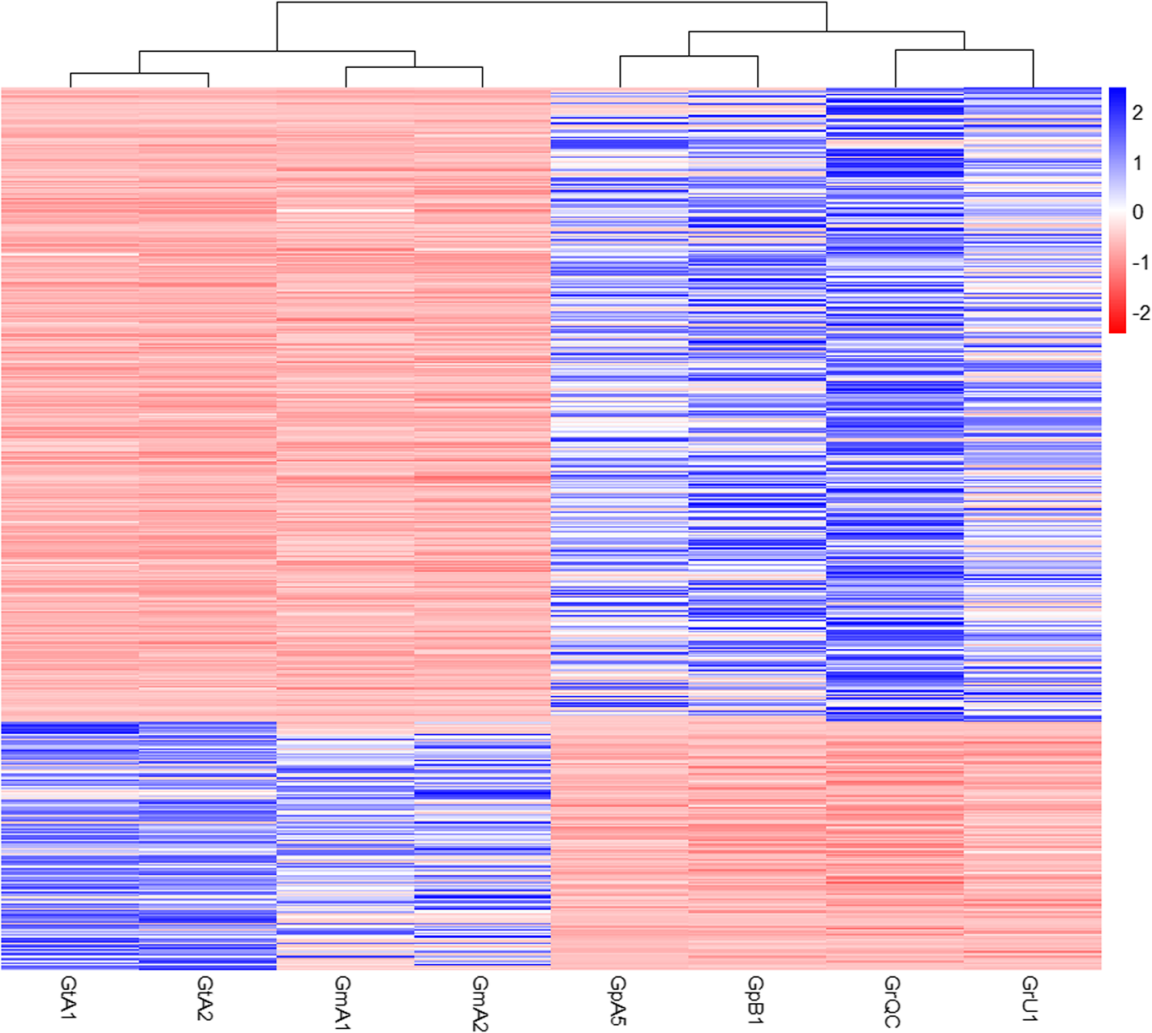
Clustering of *Globodera* species based on expression value of 545 differentially expressed genes. Differential analysis was performed by group comparison according to their pathogenicity on potato; *G. rostochiensis* and *G. pallida* populations vs. *G. mexicana* and *G. tabacum* populations (GrQC, GrU1, GpA5, GpB1 vs GtA1, GtA2, GmA1, GmA2). Expression values are score given by pheatmap function (pheatmap 1.0.10 package in R) calculated using normalized read counts, as calculated by DESEQ2

**Fig. 2:**
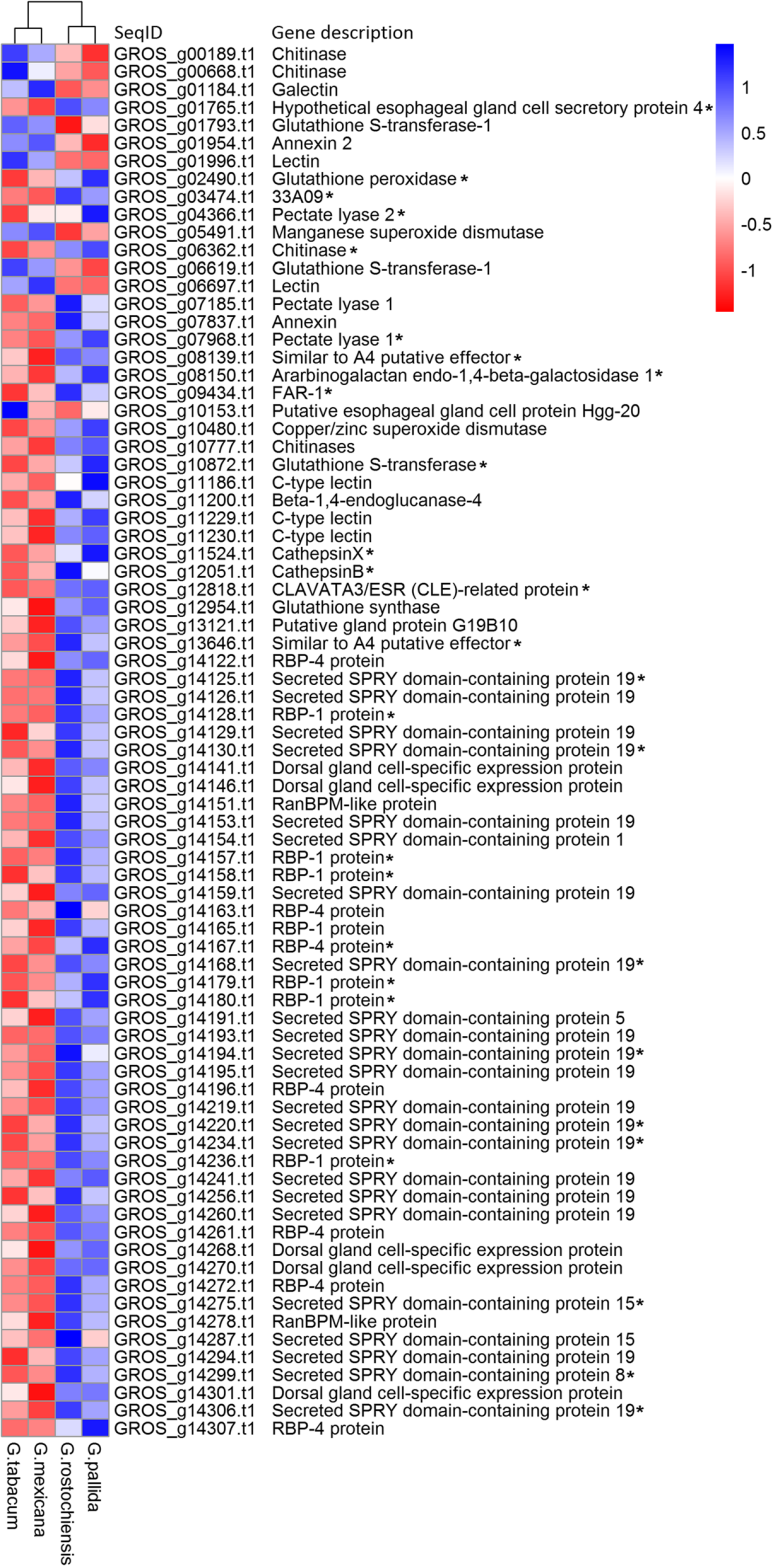
Differentially expressed effector genes between four *Globodera* species. Differential analysis was performed by group comparison according to their pathogenicity on potato; *G. rostochiensis* and *G. pallida* vs. *G. mexicana* and *G. tabacum*. Expression values are score given by pheatmap function (pheatmap 1.0.10 package in R) calculated using normalized read counts, as calculated by DESeq2. SeqID and Gene description corresponds to sequences ID and annotation of *G. rostochiensis* reference transcriptome (nGr.v1.1). The presence of a signal peptide was confirmed in the transcripts marked with an (*) but was not possible to evaluate in the others due to a truncated 5′ sequence

A total of 545 genes were found to be differentially expressed, 392 being up- and 153 down-regulated in PCN species (Fig. 1; Additional file 2: Table S1). The most differentially expressed genes were coding for a SMC (structural maintenance of chromosomes) protein and a peptidase M13 with fold changes of 30.6 and 28.5, respectively. Among DEGs, 216 were unknown genes, including 74 having a signal peptide for secretion. In the remaining 329 DEGs with known functions, 78 were known effector genes, 68 up- and 10 down-regulated in PCN populations (Fig. 2). The fold change of these effector genes varied from 26.8 (putative effector SPRY domain-containing protein 19) to 2.2 (putative dorsal gland cell-specific expression protein), with a mean fold change of 6.6 for the 68 effectors genes up-regulated in PCN populations and 3.3 for the ten genes up-regulated in non-PCN populations.

### Variant analysis

The analysis of gene polymorphism between the samples identified 1,062,443 single nucleotide polymorphisms (SNPs), 63,455 insertions or deletions (Indels) and 21,161 complex events over 1,107,386 loci. As expected, *G. rostochiensis* has the least number of variants when compared to the reference transcriptome (6%), followed by *G. tabacum* (34%), *G. mexicana* (47%) and *G. pallida* (56%). Results were very similar between the different populations of each species. Using a Bayesian inference genome scan approach, we highlighted 1181 genetic variants that were under selection in PCN species. Among them, 359 were homozygous non-synonymous variants in non-PCN populations (Additional file 3: Table S2). Ten of these genes were coding for known effectors (Table 3) and 21 genes with unknown function contained a signal peptide for secretion. The effects of these gene variations were missense variants (325), frameshift (15), conservative inframe deletion (7), disruptive inframe deletion (4), disruptive inframe insertion (3), stop gained (2), conservative inframe insertion (2) and stop codon lost (1). However, despite the presence of a variant at the same position in all populations from the same group, these variants did not always have the same impact and therefore, no frameshift, stop gained or stop codon lost were shared by all non-PCN population. For example, contig GROS_g02285.t1 has a guanine duplication (134dupG) at position 134 causing a frameshift for all populations of *G. tabacum*, whereas populations of *G. mexicana* have a SNP at the same position (134G > A) only resulting in an amino acid modification.

**Table 3:** Homozygous non-synonymous variants in effector genes found only in non-PCN species

Gene description	SeqID	Pos^1^	Ref^2^	*G. mexicana* ^3^	*G. tabacum* ^*3*^
Chorismate mutase	GROS_g02441.t1	50 66 118	His Glu GluGlu	Pro Lys LysLys	Pro Lys LysLys
Skp1	GROS_g04817.t1	84	Ala	Val	Val
Ubiquitin carboxyl-terminal hydrolase	GROS_g05177.t1	810	GlyLeu	AlaLeu	GluMet
Pectate lyase 1	GROS_g07968.t1	212	Gly	Lys	Lys
PLP synthase	GROS_g08956.t1	280	Ala	Ser	Ser
Glutathione S-transferase	GROS_g10872.t1	64	Pro	Gln	Leu
		1773	MetTrpLysPro	MetTrpLysSer	Ser
β-1,4-endoglucanase	GROS_g11200.t1	386	Ile	Lys	Lys
Putative gland protein G19B10	GROS_g13121.t1	100	ArgLeu	HisLeu	SerSer
RBP-1	GROS_g14157.t1	90	Gly	Cys	Arg
RBP-1	GROS_g14180.t1	222	GluPhe	LysSer	LysPhe

^1^ Position refers to localisation in amino acid chain

^2^ Amino acid present in the reference

^3^ Amino acid predicted different from the *G. rostochiensis* reference transcriptome

### qRT-PCR analysis

**Fig. 3:**
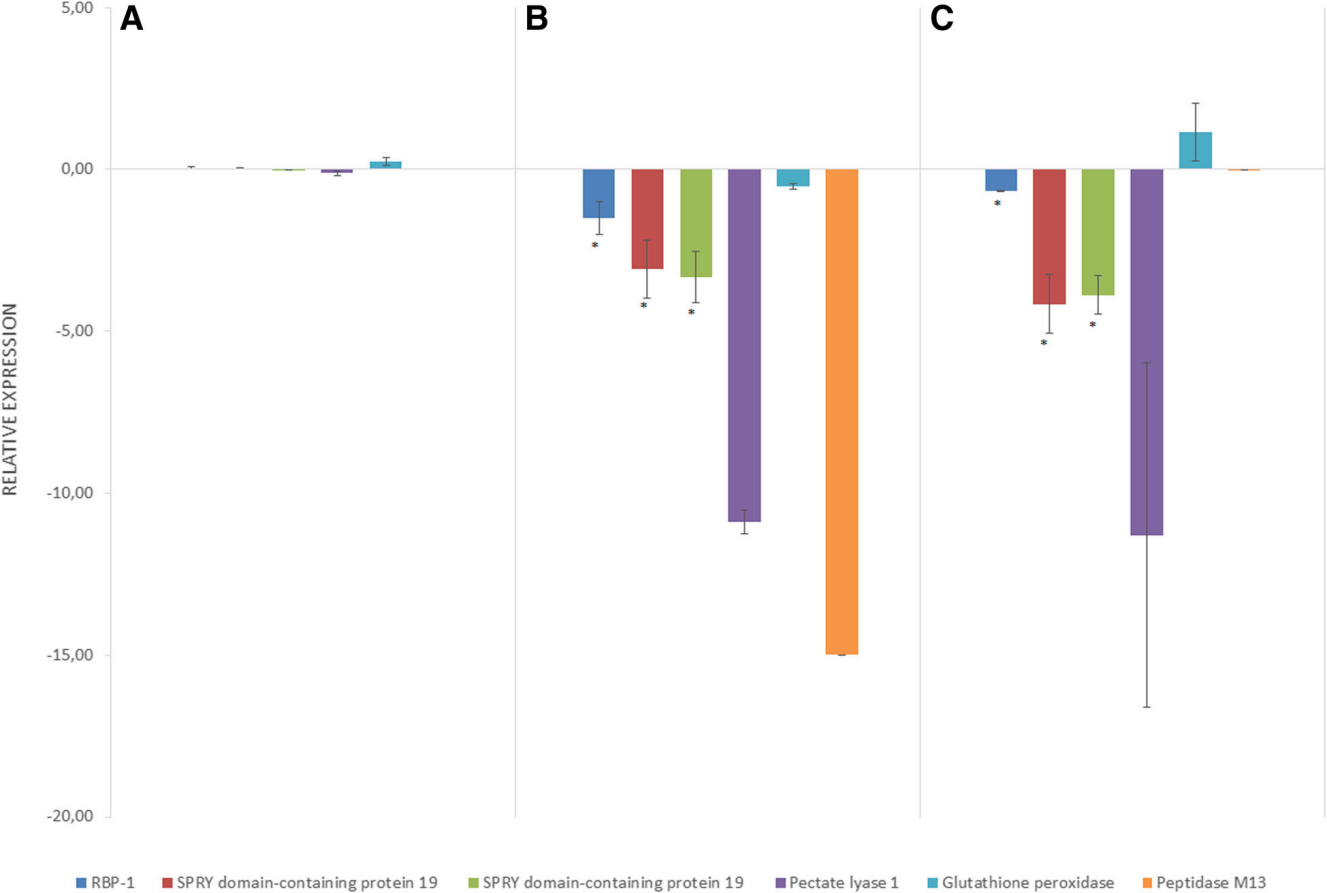
Expression of genes putatively associated with host preference in potato cyst nematodes. Change in the expression of selected effector genes after exposure to potato root diffusate in (A) potato cyst nematodes (PCN) species, *G. rostochiensis and G. pallida*, (B) non-PCN species, *G. tabacum* and *G. mexicana* or (C) PCN species exposed to tomato root diffusate. Expression was assessed by qRT-PCR and normalized using a set of reference genes (aaRS, PMP-3, and GR). The expression level of *G. rostochiensis* exposed to potato root diffusate was used as the calibrator for relative expression calculation. A default value of − 15 was assigned to samples without detectable expression. Error bars represent the standard error of the mean of each group, and significant differences are indicated by an asterisk (*) (Tukey’s test)

A subset of the differentially expressed effector genes was selected and tested by RT-qPCR to confirm their expression following exposure to root diffusate. Tested genes included RBP-1, two putative effector SPRY domain-containing protein 19, pectate lyase 1, glutathione peroxidase and Peptidase M13. When exposed to potato root diffusate, no significant difference was observed in expression levels between PCN species for all the genes tested (Fig. 3a). In non-PCN species, they were all down-regulated with a mean fold change of − 3.86, spanning from − 0.53 to − 10.88 (Fig. 3b). The qRT-PCR assay was not able to detect the presence of Peptidase M13 gene in non-PCN species, although it amplifies well on gDNA from these species (data not shown), confirming that the gene is not or very poorly expressed in non-PCN. The differences in expression of these genes between PCN and non-PCN species obtained by RT-qPCR were similar to those obtained by RNA-seq. The expression of these genes was also monitored after exposure to tomato root diffusate in order to see the effect of a different host on effector gene expression for PCN species. The expression of glutathione peroxidase and peptidase M13 remained similar to when exposed to potato root diffusate, whereas RBP-1, the two SPRY domain-containing proteins 19 and pectate lyase 1 were down-regulated (respectively − 0.67, − 4.16, − 3.88 and − 11.19) (Fig. 3c).

## Discussion

In this study, we posit that genes essential for compatible host-parasite interactions could be identified by comparing the transcriptomes of the infective stage of genetically similar *Globodera* species with different host preferences. Specifically, we investigated the ability of four *Globodera* species to infect potato. In previous studies, plant-parasitic nematodes have often been compared to free-living nematodes to identify genes involved in plant parasitism [34, 35]. Surprisingly, this resulted in the discovery of many genes that were previously never found in Metazoa (e.g. cellulase) [6]. These genes, involved in plant cell wall degradation, defense suppression, feeding site establishment, and nutrient processes, were shown to be acquired from horizontal gene transfer mainly from bacteria [6] and are now considered as an effector set, promoting the ability of plant-parasitic nematodes to grow and feed on their host. Here, we compared closely related species and searched for genes directly related to host specificity.

Understanding in detail the molecular bases of pathogenicity is a major step in plant parasitic nematology, as it is a critical turning point that can lead to the development of truly effective control methods against these pathogens. Although the host range shift between *G. tabacum* and *G. mexicana* may have occurred after their speciation (*G. tabacum* is genetically closer to *G. rostochiensis*, and *G. mexicana* to *G. pallida*), one can hypothesize that the same genetic variants should be involved, because the traits evolved from a similar genetic starting point [36]. We, therefore, assessed the changes in gene expression and gene polymorphism between potato-host and potato-non-host populations to find differences linked to host range variations, using RNA sequencing.

A total of seven predicted gene transcripts were unique to the PCN species, *G. rostochiensis* and *G. pallida*, as compared with the species non-pathogenic to potato, *G. tabacum,* and *G. mexicana*. One encodes for an ubiquitin, a protein involved in protein degradation and in regulation of gene expression when associated with histones. Several ubiquitin proteins are known to be effector proteins (e.g. Ubiquitin extension protein, Ubiquitin carboxyl-terminal hydrolase), and studies have indicated that ubiquitins probably play critical roles in plant-nematode interactions, promoting the survival of parasitic nematode [14, 37, 38]. Different ubiquitin-proteasome systems of human parasites were shown to serve as important virulence factors [39]. The other six predicted genes have unknown functions, but were all previously reported, including in other pathogens (Table 2), a result that support a putative role in parasitism. One of these genes (GROS_g12023.t1) was not amplified from the gDNA of *G. tabacum* and *G. mexicana* and presumed missing from their genome. Although no function was associated with this gene, a signature match was observed with a chromo domain. This protein structural domain is associated with chromatin organization and gene expression. This result may indicate that the loss of this gene may have impacted the expression of other genes involved in the nematode capacity to infect potato. The amplification of the other genes in the gDNA of *G. tabacum* and *G. mexicana* demonstrates that although the genes are present, they are not expressed under these conditions.

By comparing whole transcriptomic data, several genes and alleles whose expressions were correlated to the ability to parasitize the potato plant were identified. This includes 545 DEGs, among which 78 were known effector proteins (14.3%). Through the entire *G. rostochiensis* reference transcriptome (nGr.v1.1), 315 known or putative effector genes were identified based on previously published datasets [14, 37] which represent 2.2% of the 14,309 predicted genes. Thus, almost 25% of these effector genes were up-regulated in PCN species in our experiment, representing a significant enrichment (6.5 times more represented) compared to the whole transcriptome. Among the 78 differentially expressed effector genes identified, 39 were linked to microtubule cytoskeleton organization, a key element in the feeding site establishment essential to plant-parasitic nematodes. Without successfully establishing a feeding-site, the nematode fails to feed itself and dies without triggering a defense response. This corresponds to the situation observed for *G. mexicana* larvae that are able to invade the potato roots and migrate towards the vascular cylinder but fail to initiate a feeding site in potato roots. This enrichment of effector gene transcription in PCN species could be the result of a poor activation of effector gene transcription in non-PCN populations when exposed to potato root diffusate. The most significantly overexpressed DEG was a SMC_N family protein gene that was up-regulated 30.6 times in PCN populations. The SMC family proteins are involved in chromatin and DNA dynamics [40]. Furthermore, it was recently shown that one of the first genes expressed in *G. rostochiensis* and *G. pallida* following exposure to potato root diffusate was coding for a neprilysin protein, a “*transmembrane metalloprotease able to activate/inactivate peptide hormones that could be involved in a cascade of events*” [32, 41]. Interestingly, the second most highly overexpressed gene in our dataset was a peptidase M13 (GROS_g12349.t1), an unassigned homolog of the neprilysin gene, that was up-regulated 28.5 times in PCN populations. Among other up-regulated DEGs in PCN populations, 11 (3.4%) were also involved in the regulation of gene expression. The chemical signals of potato root diffusate may not allow proper activation of the infective stage of J2 larvae for non-PCN populations. Up-regulation of several regulatory genes was not observed in J2 larvae following potato root diffusates exposure, unlike PCN populations. The decreased expression of certain effector genes, when exposed to tomato root diffusate, in PCN species, as well as the increased expression of these genes in non-PCN species when exposed to tomato root diffusate, shows that the parasitic nematode may adjust its set of effectors for each potential host.

In addition, 359 non-synonymous variants showed evidence for selection between PCN and non-PCN species. Among these, ten were effector genes (Table 3) and 21 others with unknown function contained a signal peptide for excretion. These non-synonymous variants may affect the function of these effectors and pathogenicity. Also, most amino acid replacements were non-conservative, with an amino acid replacement from another side chain group, which increase the impact on protein function [42]. Several cases of polymorphism of a single amino acid having a major impact on the function of a protein have been reported, including some in these highlighted effector genes. Secreted chorismate mutase is thought to alter plant cell development, cell growth, and plant defenses and is an important virulence factor found in many plant pathogens (e.g. *Meloidogyne javanica*, *Ustilago maydis*) [43, 44]. It was shown that three single amino acid polymorphisms in *Heterodera glycines* chorismate mutase were associated with the ability to break host resistance on two particular soybean cultivars [45].

In this study, we highlighted significant differences in gene expression and gene variation between PCN (*G. rostochiensis* and *G. pallida*) and non-PCN species (*G. tabacum* and *G. mexicana*), although they are extremely close genetically. These distinctions were particularly evident in effector genes, which were highly enriched among DEGs and whose expression was reliant on the host, despite the fact that all species share a similar effector gene set in their genome, and that only a few non-synonymous variants were found in effector genes of non-PCN species. Therefore, it seems that the determinant of host specificity may reside in the regulation of essential effector gene expression. Because neprilysin was recently suggested to be involved in the early response to root diffusate and was highly overexpressed in PCN species, it might be closely implicated in pathogenicity. Ubiquitin and other genes unique to PCN, particularly those absent from non-PCN genome, also appeared as good candidates. We are strongly confident that genes responsible for the inability of non-PCN species to successfully infect potato plants are highlighted within our results and that a limited number of potential candidates have been identified.

## Conclusions

The identification of genetic particularities leading to host specificity in hyperspecialized parasites could allow designing new effective control strategies. Indeed, the inhibition of key regulators involved in host recognition may prevent the activation of infective stage and avoid substantial yield losses. Here, we showed significant differences in gene expression between PCN and non-PCN species and that host specificity could result from the regulation of a specific set of effector genes essential to parasitize specific host in closely related species. It seems that the chemical signal from root diffusate of a specific host could activate the transcription of a specific set of effectors and although we have not yet identified the exact genes involved in this regulation, we are confident that the neprilysin gene is implicated.

In future work, it would be interesting to overexpress the neprilysin gene in *G. mexicana* and *G. tabacum* to verify if this induces an up-regulation of regulator or effector genes. The identification of a single regulator gene could be used as a target for the control of PCN in a host-induced gene silencing approach.

## Methods

### *Globodera* populations

In this study, eight populations representing four species were compared. Two PCN species, *G. rostochiensis* (populations St-Amable and Netherland) and *G. pallida* (populations Chavornay and Noirmoutier) were compared to two non-PCN species *G. tabacum* (populations 75,181 and GV1) and *G. mexicana* (populations Tlaxcala and GM5) (Table 4). Twenty-five potato plants cv. Snowden and 25 tomato plants cv. MoneyMaker were grown in perlite, in 2 L containers, until they reached about 15 cm height. Root diffusate was harvested once a week, for six consecutive weeks, by the method of Fenwick [46]. Perlite was drenched with tap water until saturation and the flowing liquid was collected. The procedure was repeated two more times and the total collected liquid was filtered using milk filters (D-547, KenAG). Root diffusates were kept at 4 °C in dark plastic containers until use (< 2 months). Three hundred cysts of each population were immerged in filtered distilled water (0.2 μm Nalgene 25 mm syringe filters, Thermo Scientific) for one week and then in filtered root diffusate (0.45 μm Nalgene 25 mm syringe filters, Thermo Scientific) for three additional weeks to induce hatching of second stage larvae (J2), used for the RNA extraction.

**Table 4:** *Globodera* populations and species used in this study

Species	Potato host status	Population ID	Name and Pathotype	Origin (provider and source collection^a^)
*G. rostochiensis*	Host	GrQC	St-Amable Ro1	Canada (G. Bélair / AAFC)
		GrU1	Netherland Ro1	Netherlands (G. Smant / WUR)
*G. pallida*	Host	GpA5	Chavornay Pa3	Switzerland (G. Karssen / WUR)
		GpB1	Noirmoutier Pa2/3	France (É. Grenier / INRA IGEPP)
*G. tabacum*	Non-host	GtA1	GV1	United States (L. Miller / INRA IGEPP)
		GtA2	75,181	Mexico (L. Miller / INRA IGEPP)
*G. mexicana*	Non-host	GmA1	Tlaxcala	Mexico (D. Mugniéry / INRA IGEPP)
		GmA2	GM5	Mexico (D. Mugniéry / INRA IGEPP)

^a^Saint-Jean-sur-Richelieu Research and Development Centre, Agriculture and Agri-Food Canada (AAFC)

Department of Plant Science, Wageningen University & Research (WUR)

Institut de Génétique, Environnement et Protection des Plantes, Institut National de la Recherche Agronomique (INRA IGEPP)

### RNA extraction and sequencing

Each sample was homogenized in 650 μl lysis buffer RLT Plus (Qiagen) with a 6 mm zirconium grinding bead and 200 μL of 1 mm zirconium beads in 2 ml tubes using the PowerLyzer 24 Homogenizer (Qiagen) and stored at − 80 °C until RNA purification. Total RNA was extracted using RNeasy Mini Kit Plus (Qiagen) according to the manufacturer’s instruction and stored at − 80 °C. RNA was quantified, and its integrity assessed using a Bioanalyzer 2100 (Agilent Technologies) with the RNA 6000 Nano kit. All RNA samples had a RIN value ≥7. Libraries were generated using TruSeq Stranded mRNA Library Prep Kit (Illumina). Paired-end sequencing was done using the TruSeq SBS V3 2 × 125 bp chip on a HiSeq2500 sequencer (Illumina) at the McGill University and Genome Quebec Innovation Center in Montreal, Canada. All eight samples were multiplexed and sequenced on a single lane.

### Sequences processing

Raw reads from all populations were trim using Trimmomatic 0.36 [47] with default parameters and were mapped to the *G. rostochiensis* transcriptome (assembly version nGr.v1.1) [14] using BWA-Mem 0.7.12 with default parameters [48]. The *G. rostochiensis* transcriptome contains 14,309 putative genes. It was chosen for mapping and downstream analysis in order to work with a near complete transcriptome, to avoid contaminating sequences and because the genes providing the ability to grow on potato are theoretically included in it. To obtain up to date annotations for the reference transcriptome, we performed a conserved domain search using CD-Search 3.16 with default parameters [49]. Predicted amino acid sequences were used as an input and were obtained using AUGUSTUS 3.3 [50] with *Caenorhabditis elegans* as species parameter. In addition, sequence similarity search using NCBI [51], KEGG [52], and UniProt [53] databases were performed for unknown sequences of interest.

A phylogenetic analysis was performed using Phylogeny.fr (approximate likelihood ratio approach; bootstrap value = 100), a web-based wrapper-tool analysing phylogenetic relationships between molecular sequences [54], integrating MUSCLE 3.8.31 [55], Gblocks 0.91b [56], PhyML 3.1/3.0 (substitution model: HKY85) [57], and TreeDyn 198.3 [58]. The analysis was performed using the small subunit ribosomal RNA gene, a gene commonly used in nematode phylogenetic studies [59], and included a sequence from *C. elegans* for comparison (Accession number: NM_067514).

### Quantitative analysis

Read counts for the statistical analysis was performed using Corset 1.04 software with default parameters [60]. Statistical analysis, including normalization and differently expressed genes (DEG) identification, was made using the DEseq2 1.14.1 Bioconductor package in R [61]. The eight populations were separated into two groups according to their host/non-host status on potato for DEG identification (GrQC, GrU1, GpA5, GpB1 vs GtA1, GtA2, GmA1, GmA2) using a parametric Wald test (DE; *P* < 0.01), a normalized minimum read count of 50 for all populations and a log2 fold change (log2FC) ≥ 1.

### Variant analysis

Variant calling was done on all eight populations using Freebayes 1.0.2 software, a bayesian genetic variant detector designed to detect possible SNPs (single-nucleotide polymorphisms), indels (insertions and deletions), and complex events [62]. Analysis was done using mapping files and the reference transcriptome as input with a minimum phred score of 30 and a minimum coverage of 10. BayeScan 2.1 [63] was then used to identify loci under natural selection, using allele frequencies as input and considering the pathogenic status on potato as the principal factor for selection. The method is based on locus-specific genetic differentiation (F_ST_) outliers to detect candidate markers under selection. We relied the “plot_bayescan” function in R provided with BayeScan to calculate a posterior odds threshold (FDR = 0.05) and on a probability greater than 0.91, as this threshold indicates a strong evidence for selection [64], to select outliers associated with the pathogenicity status of the population. Three analyzes were performed, giving different random initial seed values and only outliers present in all three analyses were kept.

The impact of these genetic variations on protein structure and cellular localization was evaluated to target the variants susceptible to lead to a difference in phenotype [20, 35]. SnpEff 4.3 [65] was used to determine the impact (silent, missense or nonsenses) of the mutation while SignalP 4.1 [66] and Phobius [67] were used to predict the presence of signal peptide cleavage sites and to determine the cellular localization of the proteins.

### Validation by qPCR

Expression levels of genes of interest identified during the RNA-seq analysis were validated using qRT-PCR. Six candidate genes were chosen, based on their biological function: RBP-1 (Sequence ID: GROS_g14179.t1), putative effector SPRY domain-containing protein 19 (GROS_g14260.t1 and GROS_g14126.t1), pectate lyase 1 (GROS_g07968.t1), glutathione peroxidase (GROS_g02490.t1) and Peptidase M13 (GROS_g12349.t1). In addition, GR (glutathione reductase), PMP-3 (putative membrane transporter), and aaRS (aminoacyl tRNA synthetase) were used as a set of reference genes to normalized expression data [41]. The transcription of these genes to mRNA was quantified in J2 larvae hatched after exposure to potato root diffusate and tomato root diffusate, a compatible host for all the species under investigation, to determine if different root diffusate can affect the expression of effectors genes.

RNA extraction was performed as mentioned above. First-strand cDNA was synthesized using SuperScript II reverse transcriptase (Invitrogen, Carlsbad, California, United States) according to the manufacturer’s instructions, from 0.5 μg of total RNA and using oligo (dT)18. Three replicates were made for each treatment. Each sample was homogenized in supplied lysis buffer with a 6 mm zirconium grinding bead and 200 μL of 1 mm zirconium beads in 2 ml tubes using the PowerLyzer 24 Homogenizer (Qiagen) prior to extraction. Primers were designed using PrimerQuest tool (Integrated DNA Technologies, Inc., Coralville, Iowa, United States) based on the sequences retrieved from the *G. rostochiensis* transcriptome. Target fragments lengths were designed close to 100 bp. Primer information are listed in Table 5. Reactions were prepared using QuantiTect SYBR Green PCR kit (Qiagen) and amplified on a Mx3000P qPCR System (Agilent Technologies) for 45 cycles in a final volume of 25 μL according to the manufacturer’s instructions. Melting curve analyses were done following the amplification cycles in order to examine the specificity of the reactions. Relative expression analysis was performed using the 2^−ΔΔCt^ method [68]. *G. rostochiensis* J2 larvae exposed to potato root diffusate was used as calibrator to calculate expression fold changes for all RNA samples.

**Table 5:** Primer information

Gene	SeqID	Sequence (5′3’) forward/reverse
RBP-1	GROS_g14179.t1	GACGCCGTTTGCTTGTTCG / CTTTATTCTTGAGTTTGGTGT
SPRY domain-containing protein 19	GROS_g14260.t1	GCATTGATGGAAAGACGACAAC / GTTGCTGGTGGTTCTGATACT
SPRY domain-containing protein 19	GROS_g14126.t1	CGCGCCAAACAACAGTTAAT / GCATTTGTTCGGTCGCAAG
Pectase lyase 1	GROS_g07968.t1	GCTACTGGGTTCGGATACAA / GGCCAGATTGCGTGAAATAC
Glutathione peroxidase	GROS_g02490.t1	TCTACGACTTTGAGGTGGAAAC / GAAACGGGTTGAAGTCCAGATA
Neprilisin	GROS_g12349.t1	AATCACGCCGCCAAAGAA / CCAATGATGAGAGTGGTCGTAAA
Unknown	GROS_g09749.t1	CGCCCATCCCATTAGTGTT / CAACGACAAATCATGTTCTCCTC
Unknown	GROS_g10809.t1	AAATTCCGGTCGGCTCCT / TATTCCACGAACGGCTCCA
Polyubiquitin-B-like	GROS_g11284.t1	GCGACTGATCTTTAATGGGAAAC / CATCCTCCACGAAGACAAAGA
Unknown	GROS_g12023.t1	CGAATTGCCGGATGTTCTTG / CGTGTCAATTCGGTCGTAGAA
Unknown	GROS_g13375.t1	CGAGATGGTGTGATCAAGAAGA / TGACTGCGAGTTCGATTGG
Unknown	GROS_g13474.t1	CAGACAACACAGCACAACTTC / CTGAATCCCGGTCCTTGAAT
Unknown	GROS_g13669.t1	TTACGACTCCGCAAGTGTTC / TTGACTGCGGCGATTTCA

DNA from each species was also used to confirm the presence, in their respective genome, of seven genes for which transcripts were only observed in PCN species in the RNA-seq data. DNA extraction was performed on dry cyst using DNeasy Blood & Tissue Kits (Qiagen) according to the manufacturer’s instruction and stored at − 20 °C. Each sample was homogenized in supplied lysis buffer with a 6 mm zirconium grinding bead and 200 μL of 1 mm zirconium beads in 2 ml tubes using the PowerLyzer 24 Homogenizer (Qiagen) prior to extraction. Primer design and qPCR amplification were performed as mentioned above.

## Additional files

Additional file 1: **Figure S1.** Genetic similarities of four *Globodera* species compared to *Caenorhabditis elegans.* Phylogenetic tree of the small subunit ribosomal RNA gene sequences from *Globodera rostochiensis*, *G. pallida*, *G. mexicana*, *G. tabacum* and *C. elegans*. Analysis was performed using Phylogeny.fr, bootstrap values (*) are given next to the nodes. (PNG 15 kb)

Additional file 2: **Table S1.** Differentially expressed genes (DEG, *P* < 0.01) between PCN (*Globodera rostochiensis*, *G. pallida*) and non-PCN species (*G. tabacum*, *G. mexicana*); * mark known effector genes. SeqID and Gene description corresponds to sequences ID and annotation of *G. rostochiensis* reference transcriptome (nGr.v1.1). (XLSX 55 kb)

Additional file 3: **Table S2.** Homozygous non-synonymous predicted variants in non-PCN populations that were located on a loci under selection; * mark known effector genes. SeqID and Gene description corresponds to sequences ID and annotation of *G. rostochiensis* reference transcriptome (nGr.v1.1). (XLSX 53 kb)

## Data Availability

Sequencing data were submitted to the NCBI Sequence Read Archive under the accession number SRP146253.

## Abbreviations

aaRS: Aminoacyl tRNA synthetase
DE: Differential expression
DEG: Differentially expressed gene
GR: Glutathione reductase
Indels: Insertions/Deletions
J2 larvae: Larvae from the second juvenile stage
log2FC: Log2 Fold change
P: *P*-value
PCN: Potato cyst nematode
PMP-3: Putative membrane transporter
PPN: Plant-parasitic nematode
RIN: RNA integrity number
SMC: Structural maintenance of chromosomes
SNP: Single Nucleotide Polymorphism
