# Supplementary figures and images for: What determines host specificity in hyperspecialized plant parasitic nematodes?
Source: BMC Genomics. 2019 Jun 6;20:457. doi: 10.1186/s12864-019-5853-4 (PMC6555003; doi:10.1186/s12864-019-5853-4)

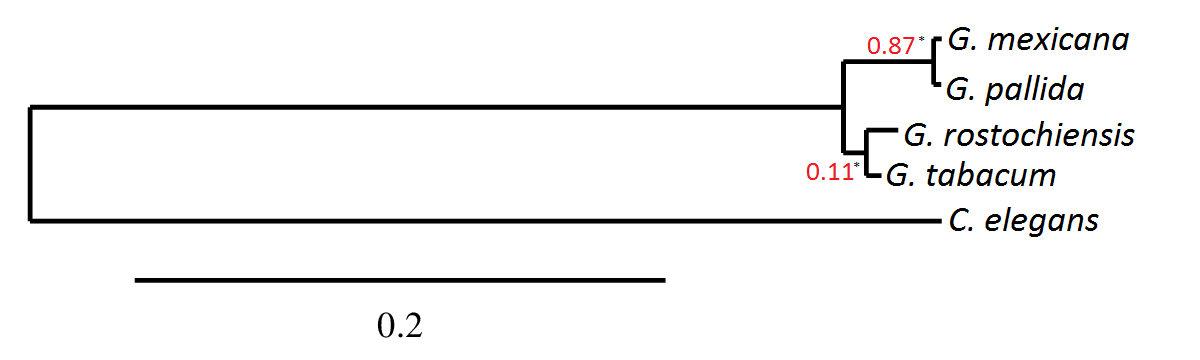

Supplement: Supplementary file 1 — Figure S1. Genetic similarities of four Globodera species compared to Caenorhabditis elegans. Phylogenetic tree of the small subunit ribosomal RNA gene sequences from Globodera rostochiensis, G. pallida, G. mexicana, G. tabacum and C. elegans. Analysis was performed using Phylogeny.fr, bootstrap values (*) are given next to the nodes. (PNG 15 kb) [file 12864_2019_5853_MOESM1_ESM.png]
